# MicroRNA-33 Deficiency Reduces the Progression of Atherosclerotic Plaque in ApoE^−/−^ Mice

**DOI:** 10.1161/JAHA.112.003376

**Published:** 2012-12-19

**Authors:** Takahiro Horie, Osamu Baba, Yasuhide Kuwabara, Yoshimasa Chujo, Shin Watanabe, Minako Kinoshita, Masahito Horiguchi, Tomoyuki Nakamura, Kazuhisa Chonabayashi, Masakatsu Hishizawa, Koji Hasegawa, Noriaki Kume, Masayuki Yokode, Toru Kita, Takeshi Kimura, Koh Ono

**Affiliations:** Department of Cardiovascular Medicine, Kyoto University, Kyoto, Japan (T.H., O.B., Y.K., Y.C., S.W., M.K., M. Horiguchi, N.K., T. Kimura, K.O.); Department of Hematology and Oncology and Clinical Innovative Medicine, Translational Research Center, Kyoto University, Kyoto, Japan; Graduate School of Medicine, Kyoto University, Kyoto, Japan; Department of Pharmacology, Kansai Medical University, Moriguchi, Osaka, Japan; Division of Translational Research, Clinical Research Institute, Kyoto Medical Center, Kyoto, 612-8555, Japan; Department of Cardiovascular Medicine, Kobe City Medical Center General Hospital, Kobe, 650-0046, Japan

**Keywords:** ABCA1, ABCG1, atherosclerosis, HDL-C, microRNA

## Abstract

**Background:**

Cholesterol efflux from cells to apolipoprotein A-I (apoA-I) acceptors via the ATP-binding cassette transporters ABCA1 and ABCG1 is thought to be central in the antiatherogenic mechanism. MicroRNA (miR)-33 is known to target ABCA1 and ABCG1 in vivo.

**Methods and Results:**

We assessed the impact of the genetic loss of miR-33 in a mouse model of atherosclerosis. MiR-33 and apoE double-knockout mice (miR-33^−/−^*Apoe*^−/−^) showed an increase in circulating HDL-C levels with enhanced cholesterol efflux capacity compared with miR-33^+/+^*Apoe*^−/−^ mice. Peritoneal macrophages from miR-33^−/−^*Apoe*^−/−^ mice showed enhanced cholesterol efflux to apoA-I and HDL-C compared with miR-33^+/+^*Apoe*^−/−^ macrophages. Consistent with these results, miR-33^−/−^*Apoe*^−/−^ mice showed reductions in plaque size and lipid content. To elucidate the roles of miR-33 in blood cells, bone marrow transplantation was performed in these mice. Mice transplanted with miR-33^−/−^*Apoe*^−/−^ bone marrow showed a significant reduction in lipid content in atherosclerotic plaque compared with mice transplanted with miR-33^+/+^*Apoe*^−/−^ bone marrow, without an elevation of HDL-C. Some of the validated targets of miR-33 such as RIP140 (NRIP1) and CROT were upregulated in miR-33^−/−^*Apoe*^−/−^ mice compared with miR-33^+/+^*Apoe*^−/−^ mice, whereas CPT1a and AMPKα were not.

**Conclusions:**

These data demonstrate that miR-33 deficiency serves to raise HDL-C, increase cholesterol efflux from macrophages via ABCA1 and ABCG1, and prevent the progression of atherosclerosis. Many genes are altered in miR-33-deficient mice, and detailed experiments are required to establish miR-33 targeting therapy in humans.

## Introduction

Although the lowering of LDL cholesterol (LDL-C) with the use of statins has revolutionized the treatment of atherosclerotic cardiovascular disease, statins can only reduce the risk of cardiovascular events by up to 50% depending on the disease status and the amount of statin used, which still leaves a large burden of residual disease risk.^1–3^ Recent studies have shown that HDL cholesterol (HDL-C) levels under statin treatment remain an independent predictor of the risk of cardiovascular events in patients who have been treated with statins to lower LDL-C levels^4^ and that HDL-C levels at baseline are a strong predictor of 12-month morbidity and mortality from cardiovascular events in patients on statins with low LDL-C levels undergoing stent placement for acute coronary syndromes.^5^ Thus, clinical and epidemiological studies have consistently shown that there is an inverse relationship between HDL-C concentration and cardiovascular risk. In fact, a 1 mg/dL (0.026 mmol/L) increment in HDL-C levels was associated with a significant decrease in the risk of coronary heart disease of 2% in men and 3% in women.^6^ All these data led us to the idea that the development of therapies that raise HDL-C is necessary to reduce cardiovascular diseases in the midst of the “statin era” in the management of atherosclerosis. Transgenic mice that overexpress apoA-I and the infusion of apoA-I/phospholipid complexes in humans are associated with reduced progression or regression of atherosclerosis.^7–9^ These observations have suggested that HDL-C-raising therapies might be an effective way to reduce the residual risk of cardiovascular diseases in patients who are being treated with current therapies.

The identification of ATP-binding cassette transporter A1 (ABCA1) as a rate-limiting factor in HDL-C biogenesis suggested that increased ABCA1 activity could inhibit atherosclerosis. Studies in mice in which the *Abca1* locus is either deleted or overexpressed generally have supported the hypothesis that ABCA1 significantly prevents atherosclerosis by maintaining circulating HDL-C levels and cellular cholesterol efflux.^10–13^ Tissue-specific knockouts of the *Abca1* locus were generated and revealed that liver and macrophage ABCA1 both play roles in preventing atherosclerosis.^14^ ABCA1 modulates cell-surface cholesterol levels, inhibits its partitioning into lipid rafts, and decreases the responsiveness of inflammatory signals from innate immune receptors. Moreover, ABCA1 has been reported to act directly as an anti-inflammatory receptor independent of its lipid transport activities.^15^ Therefore, augmentation of the function of ABCA1 might be a beneficial therapeutic approach.

MicroRNA (miR) is small, nonprotein-coding RNA that binds to specific mRNA and inhibits translation or promotes mRNA degradation. Recent reports, including ours, have indicated that miR-33 controls cholesterol homeostasis based on knockdown experiments using antisense technology.^16–18^ Moreover, antisense inhibition of miR-33 resulted in a regression of the atherosclerotic plaque volume in LDL-receptor-deficient mice.^19^ Antisense inhibition of miRNA function is an important tool for elucidating miRNA biology and evaluating its therapeutic potential. However, to determine the organ-/cell-type-specific function of miRNA over the long term in vivo, studies on miRNA-deficient mice and the analysis of specific organ/cell types from these mice are needed, especially for the development of therapeutic strategies for chronic diseases, such as atherosclerosis, dyslipidemia, and metabolic syndrome.

In the present study, we crossed miR-33-deficient mice (miR-33^−/−^) with apoE-deficient mice (*Apoe*^−/−^) to examine the impact of miR-33 deletion on the progression of atherosclerosis and demonstrated that genetic loss of miR-33 raises circulating HDL-C and decreases atherosclerotic plaque size. Furthermore, loss of leukocyte miR-33 significantly reduced the lipid content in atherosclerotic plaque. Our results indicate that miR-33 deficiency raises both HDL-C and macrophage cholesterol efflux and strongly suggest that miR-33 should be considered as a potential target for the prevention of atherosclerosis. However, some of the previously validated targets of miR-33, such as RIP140 and CROT, were upregulated in miR-33^−/−^*Apoe*^−/−^ mice compared with miR-33^+/+^*Apoe*^−/−^ mice, whereas CPT1a and AMPKα were not. Moreover, the effect of miR-33 deletion in macrophages is not as simple as the shift from the M1 to M2 phenotype reported previously.^19^ Thus, to establish that the silencing of miRNA is a therapeutic strategy for the treatment of humans, further detailed experiments are required.

## Methods

### Cell Culture and Reagents

Peritoneal macrophages were obtained from the peritoneal cavity of mice 4 days after intraperitoneal injection of 3 mL of 3% thioglycollate. For the analysis of M1/M2 markers, residual peritoneal macrophages were obtained without thioglycollate injection. The cells obtained were washed, spun at 1000 rpm for 5 minutes, and plated at a density of 5×10^5^ cells/mL with RPMI1640 medium (Nacalai Tesque, Japan) containing 10% fetal bovine serum (FBS). J774 mouse macrophages were obtained from the American Type Cell Collection (Rockville, MD) and cultured with RPMI1640 medium containing 10% FBS. The antibodies used were an anti-ABCA1 antibody (NB400-105), an anti-ABCG1 antibody (NB400-132; Novus Biologicals, Littleton, CO), an anti-GAPDH antibody (#2118S), an anti-AMPKα antibody (#2532), an anti-cleaved-caspase-3 antibody (#9661S; Cell Signaling Technology, Beverly, MA), an anti-β-actin antibody (AC-15; A5441, Sigma-Aldrich, St. Louis, MO), an anti-RIP140 antibody (sc-8997), an anti-CD3ε antibody (sc-1127; Santa Cruz, Biotechnology, CA), an anti-αSMA antibody (MO0851, Dako, Glostrup, Denmark), an anti-CROT antibody (ab103448), an anti-CPT1A antibody (ab128568), an anti-iNOS antibody (ab3523), an anti-VCAM1 antibody (ab27560), an anti-ICAM1 antibody (ab25375), an anti-IL6 antibody (ab6672), an anti-IL10 antibody (ab33471; Abcam, Cambridge, UK), an anti-single-stranded DNA (ssDNA) antibody (No. 18731; IBL, Gunma, Japan), and an anti-CD68 antibody (FA-11; Serotec, Kidlington, UK). Human acetylated LDL (acLDL) and human HDL-C were purchased from Biomedical Technologies Inc (Stoughton, MA). Anti-rabbit and anti-mouse IgG HRP-linked antibody was purchased from GE Healthcare (Amersham, UK). Human apoA-I, polyethylene glycol (PEG), ACAT inhibitor, Cpt-cAMP, Sudan IV, and oil red O were from Sigma-Aldrich. [1, 2-^3^H (N)]-Cholesterol was from Perkin Elmer (Boston, MA).

### Generation of MiR-33 and ApoE Double-Knockout Mice

To obtain miR-33 and apoE double-knockout mice (miR-33^−/−^*Apoe*^−/−^), miR-33^−/−^ mice were mated with *Apoe*^−/−^ mice, which were backcrossed to C57BL/6 mice for 10 generations.^20–22^ Because both knockout mice had a BL/6 background, miR-33^−/−^*Apoe*^−/−^ mice also had a BL/6 background. MiR-33^+/+^*Apoe*^−/−^ littermates were used as controls. After being weaned at 4 weeks of age, mice were fed normal chow (NC) containing 4.5% fat (Oriental Yeast, Tokyo, Japan) until 6 weeks of age and then switched to a Western-type diet (WTD) containing 0.15% cholesterol and 20% fat (Oriental Yeast) for 16 weeks. All the experimental protocols were approved by the Ethics Committee for Animal Experiments of Kyoto University.

### Serum Lipid Profiling

After mice were fasted for 4 to 6 hours, blood was obtained from the inferior vena cava of anesthetized mice, and serum was separated by centrifugation at 4°C and stored at −80°C. Serum lipoproteins were measured by standard methods (Accelerator selective detergent method using a Hitachi 7180 Auto Analyzer; Nagahama Life Science Laboratory, Nagahama, Japan) and HPLC methods (Sky Light Biotech, Akita, Japan).

### Western Blotting

Western blotting was performed using standard procedures as described previously.^23^ A total of 20 μg of protein was fractionated using NuPAGE 4% to 12% Bis-Tris gels (Invitrogen) and transferred to a Protran nitrocellulose transfer membrane (Whatman). The membrane was blocked using 1× phosphate-buffered saline (PBS) containing 5% nonfat milk for 1 hour and incubated with the primary antibody (anti-ABCA1, 1:1000; anti-ABCG1, 1:1000; anti–cleaved caspase-3, 1:500; anti-AMPKα, 1:1000; anti-CROT, 1:100; anti-CPT1a, 1:1000; anti-RIP140, 1:500; anti-β-actin, 1:3000; anti-GAPDH, 1:3000) overnight at 4°C. Following a washing step in PBS-0.05% Tween 20 (0.05% T-PBS), the membrane was incubated with the secondary antibody (anti-rabbit or anti-mouse IgG HRP-linked, 1:2000) for 1 hour at 4°C. The membrane was then washed in 0.05% T-PBS and detected by ECL Western Blotting Detection Reagent (GE Healthcare) using an LAS-1000 system (Fuji Film).

### RNA Extraction and Quantitative Real-Time PCR (qRT-PCR)

Total RNA was isolated and purified using TRIzol reagent (Invitrogen), and cDNA was synthesized from 1 μg of total RNA using Transcriptor First Strand cDNA Synthesis Kit (Roche) in accordance with the manufacturer's instructions. For qRT-PCR, specific genes were amplified by 40 cycles using SYBR Green PCR Master Mix (Applied Biosystems). Expression was normalized to the housekeeping gene *Actb*. Gene-specific primers are summarized as follows:

*Il6* sense, 5′ ACCACGGCCTTCCCTACTTC 3′;
*Il6* antisense, 5′ AGATTGTTTTCTGCAAGTGCATCA 3′;
*Tnf* sense, 5′ CCAGACCCTCACACTCAGATC 3′;
*Tnf* antisense, 5′ CACTTGGTGGTTTGCTACGAC 3′;
*Nos2 (Inos)* sense, 5′ GAGTCTTGGTGAAAGTGGTGTTC 3′;
*Nos2 (Inos)* antisense, 5′ TTCCCTGTCTCAGTAGCAAAGAG 3′;
*Mmp2* sense, 5′ ATCTTTGCAGGAGACAAGTTCTG 3′;
*Mmp2* antisense, 5′ TTCAGGTAATAAGCACCCTTGAA 3′;
*Serpine 1 (Pai1)* sense, 5′ TCAGCCCTTGCTTGCCTCAT 3′;
*Serpine1 (Pai1)* antisense, 5′ GCATAGCCAGCACCGAGGA 3′
*Il10* sense, 5′ AAATAAGAGCAAGGCAGTGGAG 3′;
*Il10* antisense, 5′ TCATTCATGGCCTTGTAGACAC 3′;
*Arg1* sense, 5′ AACTCTTGGGAAGACAGCAGAG 3′;
*Arg1* antisense, 5′ GTAGTCAGTCCCTGGCTTATGG 3′;
*Retnla* (*Fizz1*) sense, 5′ AGGATGCCAACTTTGAATAGGA 3′;
*Retnla* (*Fizz1*) antisense, 5′ AGTTAGCTGGATTGGCAAGAAG 3′;
*Chi3l3* (*Ym-1*) sense, 5′ CCCACCAGGAAAGTACACAGA 3′;
*Chi3l3* (*Ym-1*) antisense, 5′ CTCAGTGGCTCCTTCATTCAG 3′;
*Emr1 (F4/80)* sense, 5′ TCCAGAAGGCTCCCAAGGATA 3′;
*Emr1 (F4/80)* antisense, 5′ GGGCACTTTTGTTCTCACAGGTA 3′;
*Abca1* sense, 5′ AACAGTTTGTGGCCCTTTTG 3′;
*Abca1* antisense, 5′ AGTTCCAGGCTGGGGTACTT 3′;
*Abcg1* sense, 5′ CTGCCTCACCTCACTGTTCA 3′;
*Abcg1* antisense, 5′ TCTCGTCTGCCTTCATCCTT 3′;
*Crot* sense, 5′ TACTTTTACCACGGCCGAAC 3′;
*Crot* antisense, 5′ GACGGTCAAATCCTTTTCCA 3′;
*Cpt1a* sense, 5′ GATCTACAATTCCCCTCTGCTCT 3′;
*Cpt1a* antisense, 5′ TAGAGCCAGACCTTGAAGTAACG 3′;
*Prkaa1* (*AMPKα1*) sense, 5′ AGAGGGCCGCAATAAAAGAT 3′
*Prkaa1* (*AMPKα1*) antisense, 5′ TGTTGTACAGGCAGCTGAGG 3′;
*Nrip1* (*RIP140*) sense, 5′ AGCAGGACAAGAGTCACAGAAAC 3′;
*Nrip1* (*RIP140*) antisense, 5′ TGTGATGATTGGCAGTATCTACG 3′;
*Actb* sense, 5′ GATCTGGCACCACACCTTCT 3′;
*Actb* antisense, 5′ GGGGTGTTGAAGGTCTCAAA 3′;

### Quantitative PCR for MicroRNA

Total RNA was isolated using TRIzol reagent (Invitrogen). MiR-33 was measured in accordance with the TaqMan MicroRNA Assays (Applied Biosystems) protocol, and the products were analyzed using a thermal cycler (ABI Prism7900HT sequence detection system). Samples were normalized by U6 snRNA expression.

### Cholesterol Efflux via Mouse ApoB-Depleted Serum

Cholesterol efflux via mouse apoB-depleted serum was measured as described previously.^24,25^ Briefly, J774 cells were plated in 24 multiwell plates (7×10^4^ cells/well) and labeled for 24 hours using ^3^H-cholesterol (2 μCi/mL) in RPMI1640 plus 1% FBS. Cells were incubated in RPMI1640 containing Cpt-cAMP (0.3 mmol/L) and 0.2% BSA for an additional 16 hours to upregulate ABCA1 in J774 cells. Cells were washed and incubated for 4 hours in MEM-HEPES containing 2.8% apoB-depleted serum (equivalent to 2% serum), which was obtained after apoB lipoproteins were removed with PEG.^26^ All steps were performed in the presence of acyl-coenzyme A:cholesterol acyltransferase (ACAT) inhibitor (2 μg/mL). Cholesterol efflux was expressed as the percentage of radioactivity released from the cells in the medium relative to the total radioactivity in cells plus medium.

### Cholesterol Efflux From Mouse Peritoneal Macrophages

Cellular cholesterol efflux via apoA-I and HDL was determined as described previously.^22,27^ Briefly, thioglycollate-elicited mouse peritoneal macrophages were plated in 24-well multiplates at a density of 5×10^6^ cell/mL. Cells were cultured for 24 hours in RPMI1640 containing ^3^H-labeled acLDL (1.0 μCi/mL of ^3^H-cholesterol and 25 μg/mL of acLDL). On the next day, cells were washed 3 times with RPMI1640, then incubated for 6 hours in RPMI1640 with or without apoA-I or HDL as indicated concentrations. Cholesterol efflux was expressed as a percentage of the radioactivity released from cells in medium relative to the total radioactivity in cells plus medium.

### Flow Cytometry

Peripheral blood was collected from the orbital sinuses of 12-week-old miR-33^+/+^*Apoe*^−/−^ and miR-33^−/−^*Apoe*^−/−^ mice that were given an NC diet using heparin-coated capillary tubes. Total leukocytes were quantified from whole blood using a hematology cell counter (Celltac α MEK-6358, Nihon Kohden). Erythrocytes were lysed using a commercial RBC lysis solution (BD PharmLyse, BD Biosciences). Monocytes were identified by staining with anti-CD115–Alexa Fluor 488 antibody (eBioscience clone AFS98), and monocyte subsets were identified by staining with anti-Ly6C-APC antibody (eBioscience, clone HK1.4). Data were acquired using a BD FACSaria Flow Cytometer and analyzed with BD FACSDiva software (BD Biosciences).

### Bone Marrow Transplantation (BMT) and Assessment of Chimerism

Male mice with genotypes of miR-33^+/+^*Apoe*^−/−^ and miR33^−/−^*Apoe*^−/−^ (8 weeks old) were used as bone marrow (BM) donors. BM recipients were female miR-33^+/+^*Apoe*^−/−^ mice and miR-33^−/−^*Apoe*^−/−^ mice (8 weeks old). Thus, all the experimental mice used for BM transplantation (BMT) had an *Apoe*^−/−^ background. BM donors were euthanized by cervical dislocation, and BM cells were collected by flushing femurs and tibias with PBS supplemented with 3% FBS. The suspension was passed through 40-μm nylon mesh (Cell Strainer, BD Biosciences). Red blood cells were lysed using ACK lysing buffer (Lonza). BM cells were then washed twice with PBS supplemented with 3% FBS. To induce BM aplasia, recipients were irradiated with 2 doses of 6 Gy within an interval of 3 hours (cesium 137; Gammacell 40 Exactor) and injected intravenously with 5×10^6^ BM cells 6 hours after irradiation.^28,29^ After BMT, mice were fed NC diet for 4 weeks and then switched to a WTD for 12 weeks. At age 24 weeks, mice were euthanized and analyzed. The hematologic chimerism of mice was determined by PCR using genomic DNA from blood, BM, and tail with the PCR primers described above and by measuring miR-33 levels in BM cells by quantitative PCR at age 24 weeks (ie, 16 weeks after BMT).

### Quantification of Atherosclerosis

Atherosclerotic lesions were quantified by en face analysis of the whole aorta and by cross-sectional analysis of the proximal aorta.^30–32^ For the en face analysis of the aorta, the Sudan IV-stained aortas were photographed and used for the quantification of atherosclerotic lesions. The total aortic surface area and the lesion area were measured by image analysis (ImageJ), and the ratio of the lesion area to the total area was calculated. For the cross-sectional analysis of the aorta, the OCT-embedded aortas were sectioned using a cryostat, and 6-μm sections were obtained sequentially beginning at the aortic valve. Eight sections obtained every 24 μm from the aortic sinus were stained with oil red O and used for the quantification of lesion areas. The lesion areas of each aorta were measured using ImageJ. The average of the 8 sections from 1 mouse was taken as a value that represented the mouse.

### Immunohistochemistry

Eight sections of aortic root per mouse were stained with an anti-CD68 antibody (1:200), anti-CD3 antibody (1:50), anti-α smooth muscle actin (SMA) antibody (1:50), anti-ssDNA antibody (1:600), anti-iNOS antibody (1:200), anti-VCAM-1 antibody (1:50), anti-ICAM-1 antibody (1:50), anti-IL-6 antibody (1:400), and anti-IL-10 antibody (1:50). The lesion and positively stained areas of each aorta were measured using ImageJ. The average of the 8 sections from 1 mouse was taken as a value that represented the mouse.

### Apoptosis Assay

After being washed 3 times in PBS, peritoneal macrophages were loaded with free cholesterol (FC) by incubation with RPMI1640 containing 10% FBS supplemented with or without 300 μg/mL of acLDL plus 10 μg/mL of ACAT inhibitor (58035). The next day, cells were assayed for early- to midstage apoptosis by staining with Alexa Fluor 488–conjugated annexin V (green), as described previously^27,33,34^, using a Vybrant Apoptosis Assay Kit (Molecular Probes). Representative fields (4 to 6 fields containing ≍1000 cells) were photographed. The number of annexin V–positive cells was counted and expressed as a percentage of the total number of cells in at least 4 separate fields. For Western blotting of cleaved caspase-3, the samples were collected 48 hours after stimulation.

### Measurement of Serum ApoA-I Levels

We quantified serum apoA-I levels in mice using an ELISA assay kit for mouse apoA-I in accordance with the manufacturer's instructions (USCN Life Science Inc, Wuhan, China).

### Measurement of Total Cholesterol, Free Cholesterol, Cholesterol Ester, and Triglyceride in the Liver

Lipids in the liver were extracted by the Folch procedure^35^ and quantified using standard enzymatic colorimetric methods.

### Statistics

Data are presented as mean±SE. Statistical comparisons were performed using nonparametric analysis when the group numbers were <10 (Mann–Whitney *U* test). Otherwise, unpaired 2-tailed Student *t* tests were conducted. Statistical significance was tested by a 1-way analysis of variance with the Bonferroni post hoc test when experiments included >2 groups. The level of significance was set at a probability value of <0.05.

## Results

### MiR-33 Deficiency Reduced Atherosclerosis

**Figure 1.**
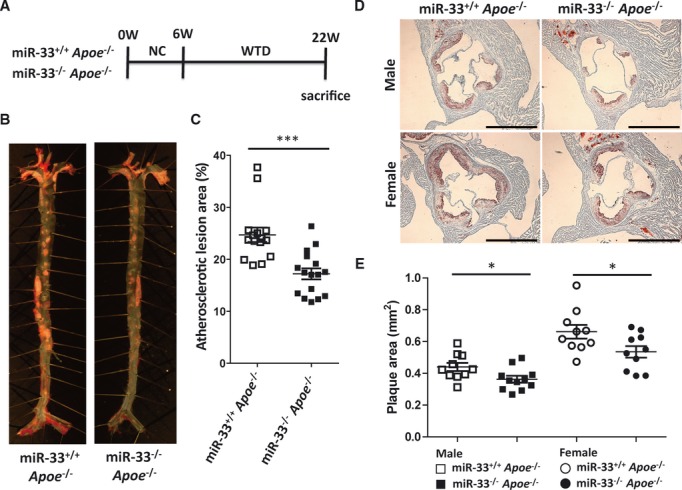
miR-33 deficiency reduced atherosclerosis. A, Experimental protocol for the analysis of atherosclerosis in miR-33^+/+^*Apoe*^*−/−*^ and miR-33^*−/−*^*Apoe*^*−/−*^ mice. B, Representative images of the en face analysis of the total aorta in miR-33^+/+^*Apoe*^*−/−*^ and miR-33^−/−^*Apoe*^−/−^ male mice. C, Quantification of the atherosclerotic lesion area in en face analysis of the total aorta in male mice. Values are mean±SE (n=15 to 16 each); ***P<0.001. D, Representative microscopic images of cross-sections of proximal aorta in miR-33^+/+^*Apoe*^−/−^ and miR-33^−/−^*Apoe*^−/−^ male and female mice. Scale bar: 1 mm. E, Quantification of the atherosclerotic plaque area in cross-sections of proximal aorta in male and female mice. Values are mean±SE (n=10 to 11 each); *P<0.05.

**Figure 2.**
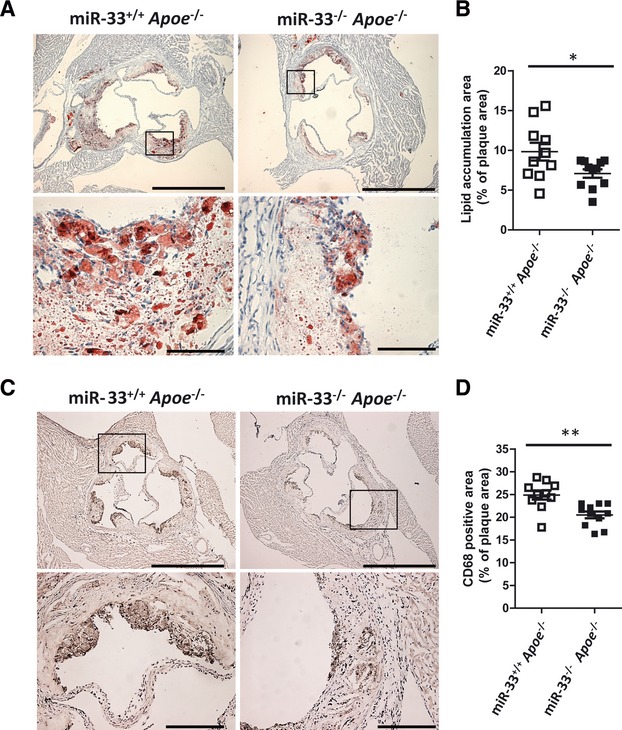
miR-33 deficiency reduced lipid accumulation and macrophage content in atherosclerotic plaque. A, Representative microscopic images of the lipid accumulation area in cross-sections of proximal aorta in miR-33^+/+^*Apoe*^−/−^ and miR-33^−/−^*Apoe*^−/−^ male mice. Scale bars: 1 mm (upper), 100 μm (lower). B, Quantification of lipid accumulation area in cross-sections of proximal aorta in male mice. Values are mean±SE (n=10 to 11 each); *P<0.05. C, Representative microscopic images of immunohistochemical staining for the macrophage marker CD68 in male mice. Scale bars: 1 mm (upper), 200 μm (lower). D, Quantification of the CD68-positive area in cross-sections of proximal aorta in male mice. Values are mean±SE (n=10 to 11 each); **P<0.01.

**Figure 3.**
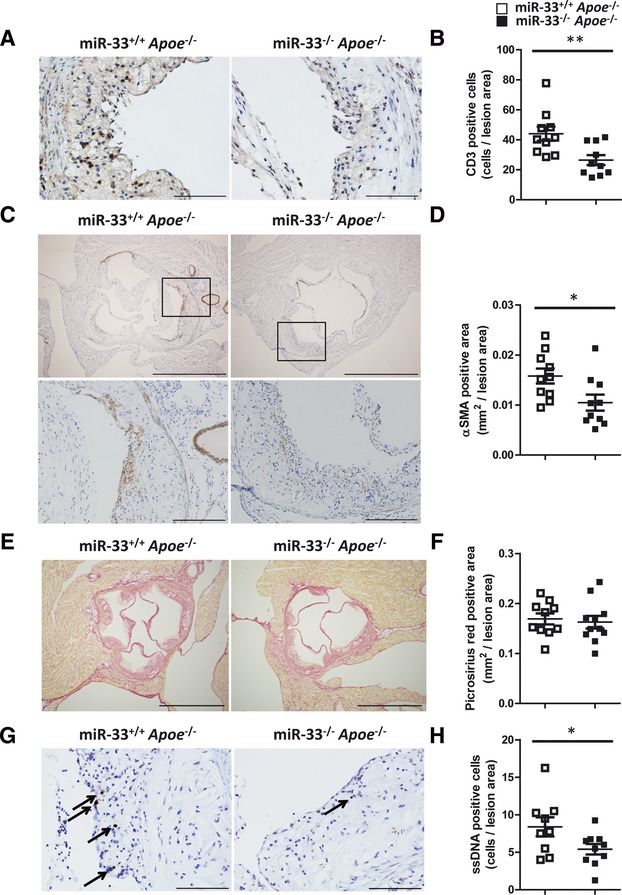
miR-33 deficiency reduced CD3-positive cell accumulation and apoptosis in atherosclerotic plaque. A, Representative microscopic images of immunohistochemical staining for the T-cell marker CD3 in male mice. Scale bar: 100 μm. B, Quantification of CD3-positive cells in cross-sections of proximal aorta in male mice. Values are mean±SE (n=10 each); **P<0.01. C, Representative microscopic images of immunohistochemical staining for αSMA in male mice. Scale bars: 1 mm (upper), 200 μm (lower). D, Quantification of the αSMA-positive area in cross-sections of proximal aorta in male mice. Values are mean±SE (n=10 each); *P<0.05. E, Representative microscopic images of picrosirius red staining for the collagen in male mice. Scale bar: 1 mm. F, Quantification of the picrosirius red–positive area in cross-sections of proximal aorta in male mice. Values are mean±SE (n=10 to 11 each). G, Representative microscopic images of immunohistochemical staining for ssDNA in male mice. Scale bar: 100 μm. H, Quantification of ssDNA-positive cells in cross-sections of proximal aorta in male mice. Values are mean±SE (n=9 to 10 each); *P<0.05.

To clarify the role of miR-33 in the progression of atherosclerosis, miR-33^−/−^ mice^22^ were mated with *Apoe*^−/-^ mice.^20,21^ Because both lines have a BL/6 background, miR-33^−/−^*Apoe*^−/−^ double-knockout mice also had a BL/6 background. The miR-33^−/−^*Apoe*^−/−^ mice were born at the expected Mendelian ratio, and miR-33^+/+^*Apoe*^−/−^ littermates were used as controls. The miR-33^+/+^*Apoe*^−/−^ and miR-33^−/−^*Apoe*^−/−^ mice were fed a WTD containing 0.15% cholesterol beginning at age 6 weeks, and atherosclerotic lesions were analyzed at age 22 weeks (Figure 1A). The atherosclerotic lesion area was examined by an en face analysis of the total aorta and cross-sections of the proximal aorta. The en face analysis of the total aorta showed that atherosclerotic lesions throughout the aorta were significantly reduced in miR-33^−/−^*Apoe*^−/−^ mice compared with miR-33^+/+^ *Apoe*^−/−^mice (male: *P*<0.001, 24.7±1.4% versus 17.2±1.1%, Figure 1B and 1C). The plaque area in the proximal aorta was extensively examined in males and females. Atherosclerotic lesions were significantly reduced in miR-33^−/−^*Apoe*^−/−^ mice of both sexes (male: *P*=0.0314, 0.44±0.025 versus 0.36±0.021 mm^2^; female: *P*=0.0372, 0.66±0.042 versus 0.54 ± 0.036 mm^2^; Figure 10D and 10E). The plaque area in females was greater than that in males. Moreover, the quantification of lipid accumulation by oil red O staining showed a significant decrease in miR-33^−/−^*Apoe*^−/−^ compared with miR-33^+/+^*Apoe*^−/−^ mice (*P*=0.034, 9.8±1.1% versus 7.1±0.5%; Figure 2A and 2B). To further analyze lesion macrophages in these mice, we stained aortic plaque using an anti-CD68 antibody. The CD68-positive area was also significantly decreased in the proximal aortas of miR-33^−/−^*Apoe*^−/−^ mice compared with miR-33^+/+^*Apoe*^−/−^ mice (*P*=0.0022, 24.9±1.0% versus 20.5±0.7%; Figure 2C and 2D). We also analyzed CD3-positive cells, αSMA-positive area, and picrosirius red staining for collagens (Figure 3A through 3F). CD3-positive cells were significantly reduced in miR-33^−/−^*Apoe*^−/−^ compared with miR-33^+/+^*Apoe*^−/−^ mice (*P*=0.0066; Figure 3A and 3B). The αSMA-positive area was also significantly reduced in miR-33^−/−^*Apoe*^−/−^ mice (*P*=0.025 Figure 3C and 3D). However, no difference was noted in the collagen area (Figure 3E and 3F). There was a significant reduction in lesional apoptosis, as measured by anti-ssDNA staining (*P*=0.0493; Figure 3G and 3H). These results indicated that a deficiency of miR-33 decreased atherosclerotic plaque size and lipid content and reduced the accumulation of macrophages and T cells in atherosclerotic plaques.

### MiR-33 Deficiency Increased HDL-C

**Figure 4.**
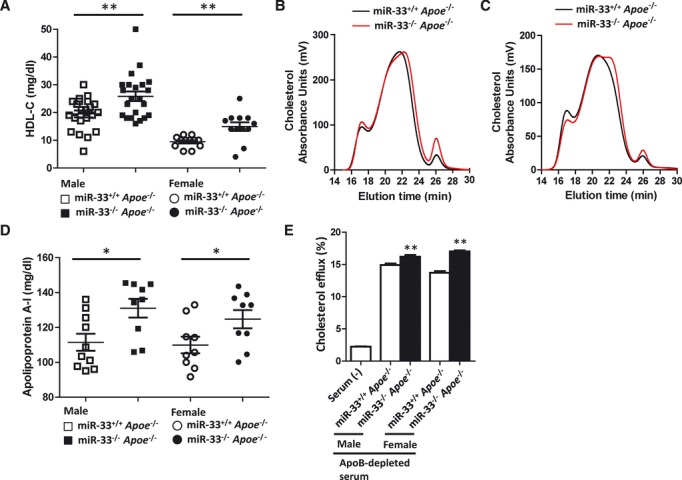
miR-33 deficiency increased HDL-C. A, Serum HDL-C levels determined by standard methods in miR-33^+/+^*Apoe*^−/−^ and miR-33^−/−^*Apoe*^−/−^ male and female mice. Values are mean±SE (male, n=22; female, n=11 each); **P<0.01. B, Representative HPLC analysis of serum cholesterol from male miR-33^+/+^*Apoe*^−/−^ and miR-33^−/−^*Apoe*^−/−^ mice. C, Representative HPLC analysis of serum cholesterol from female miR-33^+/+^*Apoe*^−/−^ and miR-33^−/−^*Apoe*^−/−^ mice. D, Serum apoA-I levels in miR-33^+/+^*Apoe*^−/−^ and miR-33^−/−^*Apoe*^−/−^ mice; *P<0.05. E, Cholesterol efflux via apoB-depleted serum from miR-33^+/+^*Apoe*^−/−^ and miR-33^−/−^*Apoe*^−/−^ mice using ^3^H-cholesterol-labeled J774 mouse macrophages. Values are mean±SE (n=6 each); **P<0.01. HDL-C indicates high-density lipoprotein cholesterol; HPLC, high-performance liquid chromatography.

We previously reported that miR-33^−/−^ mice showed 22% to 39% higher serum HDL-C levels than wild-type mice.^22^ We measured HDL-C levels in the serum of miR-33^+/+^*Apoe*^−/−^ and miR-33^−/−^*Apoe*^−/−^ mice at the time of euthanization by the standard method. HDL-C was significantly elevated in miR-33^−/−^ *Apoe*^−/−^ mice compared with miR-33^+/+^*Apoe*^−/−^ mice of both sexes (Figure 4A). We further classified and quantified serum lipoproteins by high-performance liquid chromatography (HPLC). Representative results of the HPLC elution profile of serum of both sexes are shown in Figure 4B and 4C, and lipid profiles are summarized in Table 1. These results show that only the HDL-C level differed between the serum of miR-33^+/+^*Apoe*^−/−^ and miR-33^−/−^*Apoe*^−/−^ mice. Serum apoA-I levels are shown in Figure 4D. To assess the cholesterol efflux capacity of the serum, cholesterol efflux via apolipoprotein B (apoB)–depleted serum was measured using ^3^H-cholesterol-labeled J774 mouse macrophages. The mean values of serum HDL-C used in this experiment were 17.8±1.4 versus 19.5±1.8 mg/dL in males and 10.2±0.5 versus 17.3±1.7 mg/dL in females (miR-33^+/+^ *Apoe*^−/−^ versus miR-33^−/−^*Apoe*^−/−^ mice, n=6 for each group). ApoB-depleted serum from miR-33^−/−^*Apoe*^−/−^ mice significantly promoted cholesterol efflux in J774 macrophages (Figure 4E). These results indicated that deficiency of miR-33 elevated serum cholesterol efflux capacity, possibly through the elevation of HDL-C levels.

**Table 1.** Serum Lipid Profiling of MiR-33^+/+^*Apoe*^−/−^ and MiR-33^−/−^*Apoe*^−/−^ Mice by HPLC

	Lipoprotein		Male		Female	
			
	Major (Fraction No.) Diameter	Subclass (Fraction No.)	miR-33^+/+^*Apoe*^−/−^	miR-33^−/−^*Apoe*^−/−^	miR-33^+/+^*Apoe*^−/−^	miR-33^−/−^*Apoe*^−/−^
TC, mg/dL			705.3±72.1	768.4±45.2	598.9±88.8	574.2±70.0

	CM (1 to 2), >80 nm		129.2±21.6	157.9±19.8	124.6±20.9	138.6±28.11

	VLDL (3 to 7), 30 to 80 nm		419.5±44.1	450.9±30.0	353.6±51.7	322.1±36.4

		Large VLDL (3 to 5)	279.3±32.2	316.4±24.9	245.1±35.1	230.2±29.5

		Medium VLDL (6)	100.5±9.8	96.77±5.67	79.04±12.17	66.37±6.15

		Small VLDL (7)	39.74±3.31	37.75±1.47	29.47±4.87	25.6±2.12

	LDL (8 to 13), 16 to 30 nm		133.8±14.9	126.4±5.23	103.0±16.2	90.81±6.72

		Large LDL (8)	47.47±4.40	44.05±1.53	36.58±6.03	31.60±2.61

		Medium LDL (9)	39.93±4.56	36.35±1.71	31.99±5.26	27.53±2.11

		Small LDL (10)	25.63±3.42	24.72±1.49	19.78±3.05	17.36±1.23

		Very small LDL (11 to 13)	20.75±2.75	21.25±1.19	14.65±1.89	14.33±1.27

	HDL (14 to 20), 8 to 16 nm		22.79±1.28	33.21±2.51**	17.62±0.73	22.71±2.52*

		Very large HDL (14 to 15)	3.04±0.39	3.66±0.17	3.57±0.42	3.89±0.56

		Large HDL (16)	5.88±0.63	9.39±0.87**	4.00±0.28	5.29±0.47*

		Medium HDL (17)	8.04±0.71	11.88±1.21*	4.49±0.36	6.79±0.26**

		Small HDL (18)	2.61±0.12	3.73±0.30**	2.16±0.13	2.87±0.36

		Very small HDL (19 to 20)	3.23±0.25	4.55±0.27**	3.40±0.45	3.87±0.61

TG, mg/dL			39.94±8.29	48.32±10.14	17.60±1.44	24.75±5.19

Values are mean±SEM. After a 4-hour fast, blood was obtained from mice fed a WTD for 16 weeks (male, n=6 each; female, n=5 each). The serum was analyzed by HPLC, as described in Methods. TC indicates total cholesterol; TG, triglyceride; CM, chylomicrons; VLDL, very low-density lipoprotein; LDL, low-density lipoprotein; HDL, high-density lipoprotein.

* *P*<0.05

** *P*<0.01 compared with miR-33^+/+^*Apoe*^−/−^ mice.

### Peritoneal Macrophages From MiR-33^−/−^*Apoe*^−/−^ Mice Showed Improved Cholesterol Efflux

**Figure 5.**
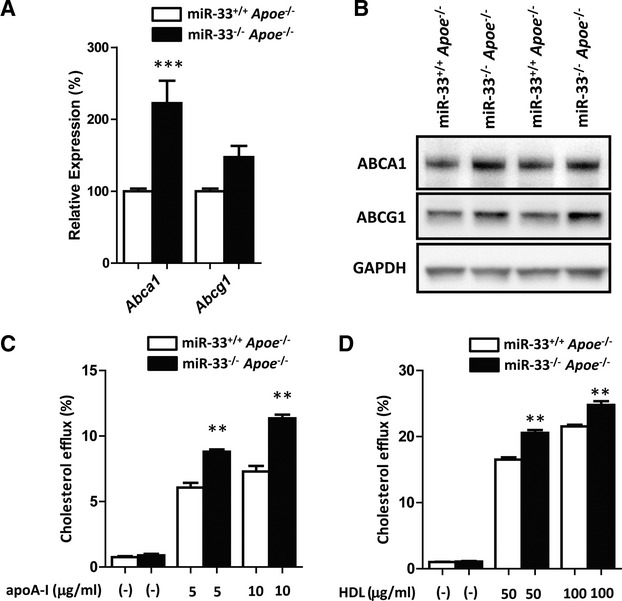
miR-33 deficiency improved cholesterol efflux in macrophages. A, Quantitative real-time PCR analysis of *Abca1* and *Abcg1* in macrophages from miR-33^+/+^*Apoe*^−/−^ and miR-33^−/−^*Apoe*^−/−^ mice. Values from miR-33^+/+^*Apoe*^−/−^ were set at 100%. Values are mean±SE (n=7 each); ***P<0.001. B, Western blotting analysis of ABCA1 and ABCG1 in thioglycollate-elicited peritoneal macrophages from miR-33^+/+^*Apoe*^−/−^ and miR-33^−/−^*Apoe*^−/−^ mice. GAPDH was used as a loading control. C, Cholesterol efflux from thioglycollate-elicited peritoneal macrophages in the presence or absence of apoA-I (5 or 10 μg/mL). Values are mean±SE (n=6 each); **P<0.01. D, Cholesterol efflux from thioglycollate-elicited peritoneal macrophages in the presence or absence of HDL-C (50 or 100 μg/mL). Values are mean±SE (n=6 each); **P<0.01.

To characterize the function of macrophages in cholesterol efflux, thioglycollate-elicited peritoneal macrophages (PEMs) were isolated from mice. Previously, we and others have shown that miR-33 targeted the 3′ untranslated region (UTR) of *Abca1* and *Abcg1*.^16–18,22^ mRNA expression of ABCA1 and protein expression of ABCA1 and ABCG1 were significantly increased in PEMs from miR-33^−/−^*Apoe*^−/−^ mice compared with PEMs from miR-33^+/+^*Apoe*^−/−^ mice (Figure 5A and 5B). To test the hypothesis that enhanced cholesterol efflux in macrophages contributed to the reduction in the development of atherosclerosis in miR-33^−/−^*Apoe*^−/−^ mice, cholesterol efflux to apoA-I and HDL-C was measured using ^3^H-cholesterol-labeled acetylated low-density lipoprotein (acLDL). Both apoA-I- and HDL-C-mediated cholesterol efflux were significantly elevated in macrophages from miR-33^−/−^*Apoe*^−/−^ compared with macrophages from miR-33^+/+^*Apoe*^−/−^ mice in a dose-dependent manner (Figure 5C and 5D). These results indicate that miR-33 deficiency improved macrophage cholesterol efflux by increasing the expressions of macrophage ABCA1 and ABCG1.

### MiR-33 Deficiency Affected Circulating Monocytes and Lesional Macrophages

**Figure 6.**
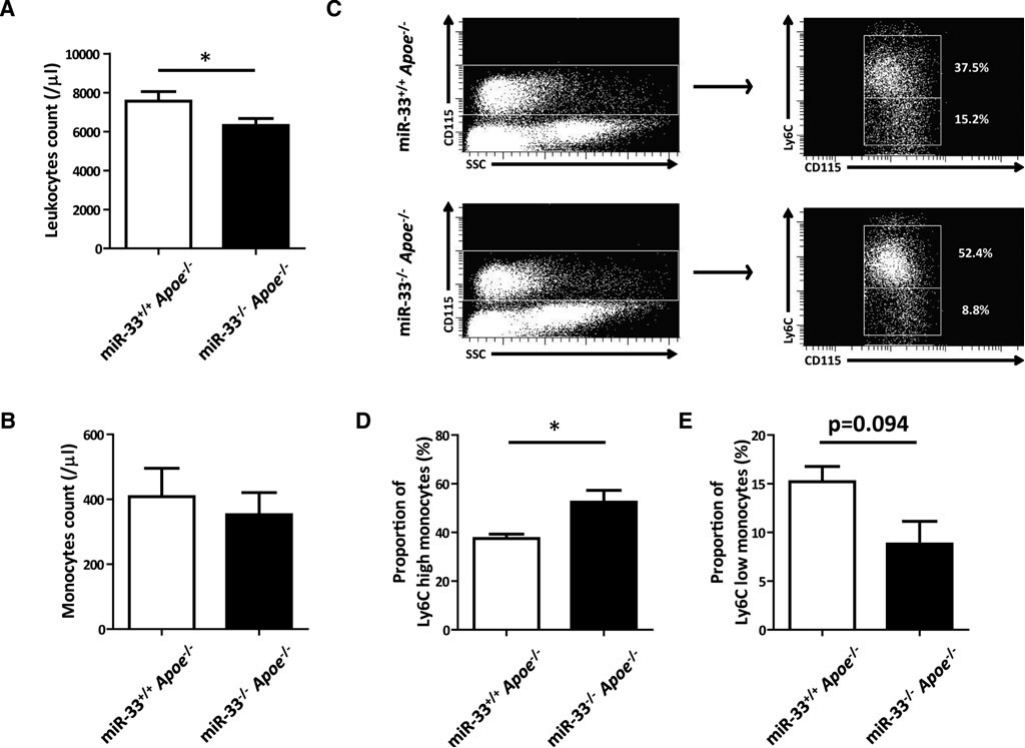
miR-33^−/−^*Apoe*^−/−^ mice had reduced leukocyte numbers in peripheral blood and a greater Ly-6C^high^ monocyte subset compared with miR-33^+/+^*Apoe*^−/−^ mice. A, Leukocyte count in peripheral blood in miR-33^+/+^*Apoe*^−/−^ and miR-33^−/−^*Apoe*^−/−^ mice. Values are mean±SE (n=15 to 16 each); *P<0.05. B, Numbers of monocyte in peripheral blood in miR-33^+/+^*Apoe*^−/−^ and miR-33^−/−^*Apoe*^−/−^ mice. Values are mean±SE (n=9 each). C, Scheme for gating of monocytes using an anti-CD115 antibody, and representative dot plots showing the quantification of Ly-6C^high^ and Ly-6C^low^ monocyte subsets in miR-33^+/+^*Apoe*^−/−^ and miR-33^−/−^*Apoe*^−/−^ mice. D, Proportion of the Ly-6C^high^ monocyte subset to total monocytes in miR-33^+/+^*Apoe*^−/−^ and miR-33^−/−^*Apoe*^−/−^ mice. Values are mean±SE (n=9 each.); *P<0.05. E, Proportion of the Ly-6C^low^ monocyte subset to total monocytes in miR-33^+/+^*Apoe*^−/−^ and miR-33^−/−^*Apoe*^−/−^ mice. Values are mean±SE (n=9 each).

**Figure 7.**
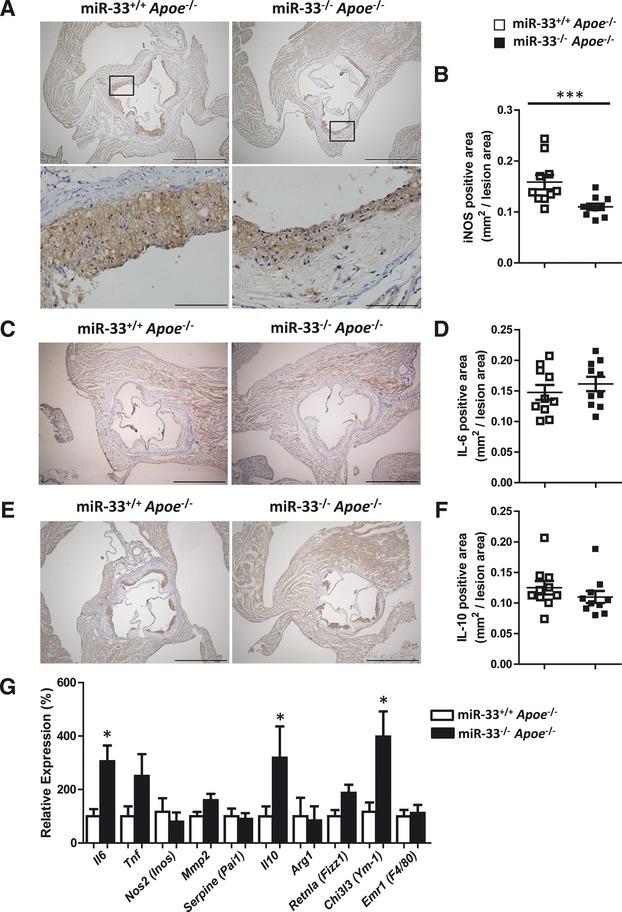
miR-33 deficiency reduced the iNOS-positive areas in atherosclerotic plaque and induced coordinated M1 and M2 marker expression in PEMs. A, Representative microscopic images of immunohistochemical staining for iNOS in male mice. Scale bars: 1 mm (upper), 100 μm (lower). B, Quantification of the iNOS-positive area in cross-sections of proximal aorta in male mice. Values are mean±SE (n=10 each); ***P<0.001. C, Representative microscopic images of immunohistochemical staining for IL-6 in male mice. Scale bar: 1 mm. D, Quantification of the IL-6-positive area in cross-sections of proximal aorta in male mice. Values are mean±SE (n=10 each). E, Representative microscopic images of immunohistochemical staining for IL-10 in male mice. Scale bar: 1 mm. F, Quantification of the IL-10-positive area in cross-sections of proximal aorta in male mice. Values are mean±SE (n=10 each). G, Quantitative real-time PCR analysis of proinflammatory (M1) and anti-inflammatory (M2) markers in residual PEMs from miR-33^+/+^*Apoe*^−/−^ and miR-33^−/−^*Apoe*^−/−^ mice. Values from miR-33^+/+^*Apoe*^−/−^ mice were set at 100%. Values are mean±SE (n=7 each); *P<0.05.

**Figure 8.**
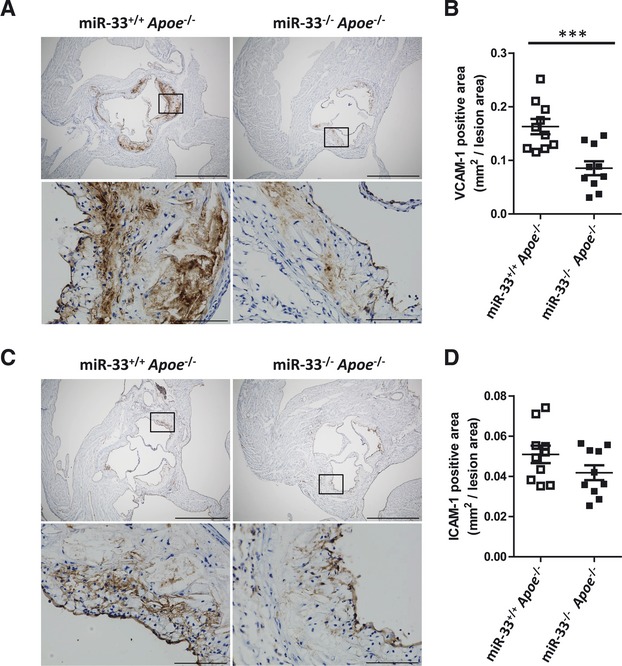
miR-33 deficiency reduced the VCAM-1-positive area in atherosclerotic plaque. A, Representative microscopic images of immunohistochemical staining for VCAM-1 in male mice. Scale bars: 1 mm (upper), 100 μm (lower). B, Quantification of the VCAM-1-positive area in cross-sections of proximal aorta in male mice. Values are mean±SE (n=10 each); ***P<0.001. C, Representative microscopic images of immunohistochemical staining for ICAM-1 in male mice. Scale bars: 1 mm (upper), 100 μm (lower). D, Quantification of the ICAM-1-positive area in cross-sections of proximal aorta in male mice. Values are mean±SE (n=10); *P*=0.127.

Our findings thus far indicate that miR-33 deficiency reduced the accumulation of inflammatory cells in atherosclerotic plaque. We then examined whether miR-33 deficiency influenced the monocyte count or subset frequency in peripheral blood. The total leukocyte count in miR-33^−/−^*Apoe*^−/−^ mice was significantly less than that in miR-33^+/+^*Apoe*^−/−^ mice (Figure 6A). Blood monocyte subsets were discriminated by flow cytometry on the basis of their expression of CD115 and Ly6C.^36^ The frequency of proinflammatory Ly6C^high^ monocytes in miR-33^−/−^*Apoe*^−/−^ mice was significantly higher than that in miR-33^+/+^*Apoe*^−/−^ mice (Figure 6B through 6E). We further measured iNOS, interleukin (IL)–6, and IL-10 expression in atherosclerotic plaque by immunohistochemistry. As shown in Figure 7A and 7B, the iNOS-positive area in atherosclerotic plaque in miR-33^−/−^*Apoe*^−/−^ mice was significantly less than that in miR-33^+/+^*Apoe*^−/−^ mice (*P*=0.0057). On the other hand, no differences were observed in IL-6 and IL-10 immunostaining (Figure 7C through 7F). We also characterized miR-33^−/−^*Apoe*^−/−^ macrophages by analyzing the expression of classically activated or proinflammatory (M1) and alternatively activated or anti-inflammatory (M2) macrophage markers using mouse PEMs. A previous article indicated that the inhibition of miR-33 by antisense oligonucleotide enhanced M2 marker expression in macrophages.^19^ However, our experiments indicated that the mRNA levels of both M2 markers such as *IL-10* and *Chi3l3* mRNA and an M1 marker such as *IL-6* in PEMs from miR-33^−/−^*Apoe*^−/−^ mice were significantly elevated compared with those from miR-33^+/+^*Apoe*^−/−^ mice (Figure 7G). Overall, these results demonstrate that a loss of miR-33 may have affected multiple pathways in both pro- and anti-inflammatory processes. Moreover, we analyzed adhesion molecule expression by immunostaining. As shown in Figure 8A and 8B, the VCAM-1-positive area in atherosclerotic plaque in miR-33^−/−^*Apoe*^−/−^ was significantly less than that in miR-33^+/+^*Apoe*^−/−^ mice (*P*=0.0008). The ICAM-1-positive area in miR-33^−/−^*Apoe*^−/−^ mice tended to be less than that in miR-33^+/+^*Apoe*^−/−^ mice (*P*=0.127), shown in Figure 8C and 8D.

### MiR-33 Target Genes Were Altered in the Livers of MiR-33^−/−^ Mice on an *Apoe*^−/−^ Background

**Figure 9.**
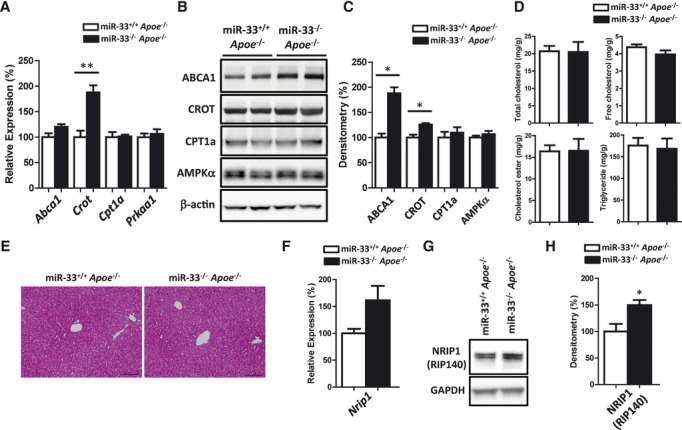
Expression of ABCA1 and CROT in livers and RIP140 in macrophages is elevated in miR-33^−/−^*Apoe*^−/−^ mice compared with miR-33^+/+^*Apoe*^−/−^ mice. A, Quantitative real-time PCR analysis of *Abca1*, *Crot*, *Cpt1a*, and *Prkaa1*in livers from miR-33^+/+^*Apoe*^−/−^ and miR-33^−/−^*Apoe*^−/−^ mice. Values from miR-33^+/+^*Apoe*^−/−^ mice were set at 100%. Values are mean±SE (n=9 to 11each); **P<0.01. B, Western analysis of ABCA1, CROT, CPT1a, and AMPKα in livers from miR-33^+/+^*Apoe*^−/−^ and miR-33^−/−^*Apoe*^−/−^ mice. β-actin was used as a loading control. C, Densitometry of ABCA1, CROT, CPT1a, and AMPKα in livers from miR-33^+/+^*Apoe*^−/−^ and miR-33^−/−^*Apoe*^−/−^ mice. Values from miR-33^+/+^*Apoe*^−/−^ mice were set at 100%. Values are mean±SE (n=4 each); *P<0.05. D, Total cholesterol, free cholesterol, cholesterol ester, and triglyceride levels in livers of miR-33^+/+^*Apoe*^−/−^ and miR-33^−/−^*Apoe*^−/−^ mice. Values are mean±SE (n=9 to 11 each). E, HE staining of livers of miR-33^+/+^*Apoe*^−/−^ and miR-33^−/−^*Apoe*^−/−^ mice at age 20 weeks fed NC. Scale bar: 100 μm. F, Quantitative real-time PCR analysis of *Nrip1* (RIP140) in peritoneal macrophages from miR-33^+/+^*Apoe*^−/−^ and miR-33^−/−^*Apoe*^−/−^ mice. Values from miR-33^+/+^*Apoe*^−/−^ mice were set at 100%. Values are mean±SE (n=7 each). G, Western analysis of NRIP1 (RIP140) in peritoneal macrophages from miR-33^+/+^*Apoe*^−/−^ and miR-33^−/−^*Apoe*^−/−^ mice. GAPDH was used as a loading control. H, Densitometry of NRIP1 (RIP140) in peritoneal macrophages from miR-33^+/+^*Apoe*^−/−^ and miR-33^−/−^*Apoe*^−/−^ mice. Values from miR-33^+/+^*Apoe*^−/−^ mice were set at 100%. Values are mean±SE (n=4 each); *P<0.05.

It has already been shown that miR-33 targets several genes that affect cholesterol and fatty acid synthesis. We measured the mRNA and protein levels of ABCA1, CROT, and CPT1a in the livers of these mice. As shown in Figure 9A through 9C, mRNA of CROT and protein levels of ABCA1 and CROT in miR-33^−/−^*Apoe*^−/−^ mice were significantly elevated compared with those in miR-33^+/+^*Apoe*^−/−^ mice. No differences were observed in CPT1a and AMPKα expressions. Next, we measured the lipid content in the liver. However, there was no difference in total cholesterol, free cholesterol, cholesterol ester, or triglyceride levels in the livers of miR-33^+/+^*Apoe*^−/−^ mice and miR-33^−/−^*Apoe*^−/−^ mice (Figure 9D). No apparent changes in histology were observed in the livers of these mice, as shown by HE staining (Figure 9E). Moreover, we measured the level of RIP140 (NRIP1), which has been shown to be one of the targets of miR-33 in macrophages of these mice.^37^ Protein level of RIP140 in miR-33^−/−^*Apoe*^−/−^ macrophages was significantly increased compared with that in miR-33^+/+^*Apoe*^−/−^ macrophages (Figure 9F through 9H), which may be one of the reasons why the expression of inflammatory cytokine such as IL-6 in PEMs of miR-33^−/−^*Apoe*^−/−^ mice was increased compared with that in miR-33^+/+^*Apoe*^−/−^ mice.^38^

### Loss of MiR-33 in Blood Cells Did Not Alter Serum HDL-C Levels

**Figure 10.**
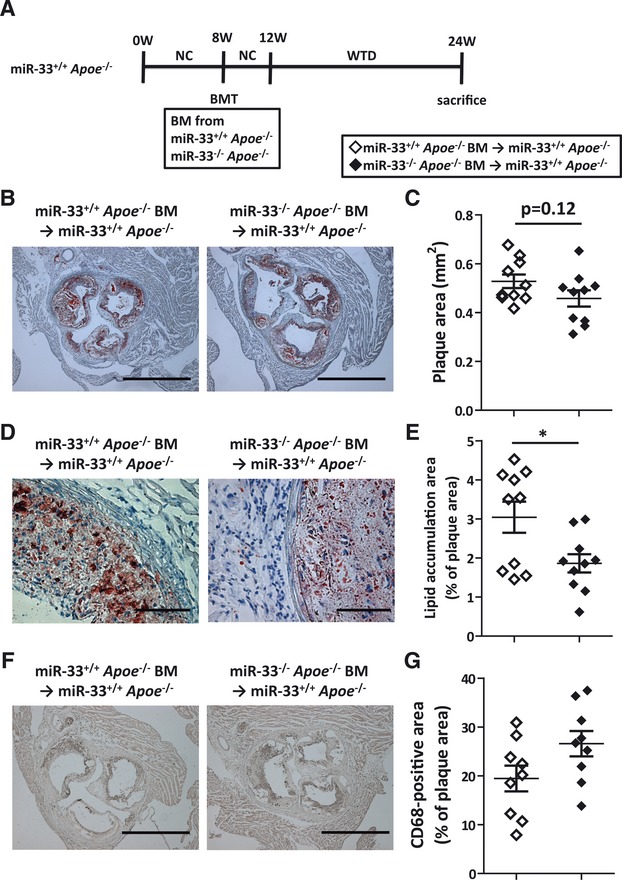
Lipid accumulation area in atherosclerotic lesions was reduced in miR-33^+/+^*Apoe*^−/−^ mice transplanted with miR-33^−/−^*Apoe*^−/−^ BM. A, Experimental protocol for bone marrow transplantation from miR-33^+/+^*Apoe*^−/−^ and miR-33^−/−^*Apoe*^−/−^ mice to miR-33^+/+^*Apoe*^−/−^ mice. B, Representative microscopic images of cross-sections of proximal aorta in mice transplanted with miR-33^+/+^*Apoe*^−/−^ and miR-33^−/−^*Apoe*^−/−^ BM. Scale bar: 1 mm. C, Quantification of the atherosclerotic plaque area in cross-sections of proximal aorta. Values are mean±SE (n=10 each). D, Representative microscopic images of the lipid accumulation area in atherosclerotic lesions in mice transplanted with miR-33^+/+^*Apoe*^−/−^ and miR-33^−/−^*Apoe*^−/−^ BM. Scale bar: 100 μm. E, Quantification of the lipid accumulation area in cross-sections of proximal aorta. Values are mean±SE (n=10 each); *P<0.05. F, Representative microscopic images of immunohistochemical staining for the macrophage marker CD68 in mice transplanted with miR-33^+/+^*Apoe*^−/−^ and miR-33^−/−^*Apoe*^−/−^ BM. Scale bar: 1 mm. G, Quantification of CD68-positive area in cross-sections of proximal aorta. Values are mean±SE (n=9 each). BM indicates bone marrow.

The results of these experiments show that both the rise in HDL-C level and the improvement in macrophage cholesterol efflux may have contributed to the reduction in atherosclerotic plaques. To elucidate the contribution of miR-33 in macrophages to the development of atherosclerosis in vivo, we used bone marrow transplantation (BMT) to generate *Apoe*^−/−^ mice selectively deficient in leukocyte miR-33. Male mice with miR-33^+/+^*Apoe*^−/−^ or miR33^−/−^*Apoe*^−/−^ genotypes (8 weeks old) were used as bone marrow (BM) donors. BM recipients were female miR-33^+/+^*Apoe*^−/−^ mice (8 weeks old). Thus, all the mice used for BMT had an *Apoe*^−/−^ background. After BMT, mice were fed NC for 4 weeks and then switched to a WTD for 12 weeks. At age 24 weeks, mice were euthanized and analyzed (Figure 10A). Successful hematopoietic reconstitution after BMT was confirmed by PCR amplification of the whole-blood genome 4 weeks after BMT and by PCR amplification of the BM and tail genomes at the time of euthanization. MiR-33 was barely detectable in BM cells from miR-33^−/−^*Apoe*^−/−^ BM recipients by quantitative PCR analysis for miR-33 (data not shown). The serum lipid profile of recipient mice is shown in Table 2. Serum HDL-C levels of miR-33^+/+^*Apoe*^−/−^ BM recipients were the same as those in miR-33^−/−^*Apoe*^−/−^ BM recipients, which were similar to the levels in miR-33^+/+^*Apoe*^−/−^ mice in Figure 4A. These results indicated that miR-33 expression in macrophages did not contribute to serum HDL-C levels, which is consistent with the results that the liver and intestine are the major sources of HDL-C.^39,40^

**Table 2.** Serum Lipid Profiling of MiR-33^+/+^*Apoe*^−/−^ Mice Transplanted With MiR-33^+/+^*Apoe*^−/−^ and MiR-33^−/−^*Apoe*^−/−^ BM by Standard Method

	TC, mg/dL	HDL-C, mg/dL	LDL-C, mg/dL	TG, mg/dL
miR-33^+/+^*Apoe*^−/−^ BM recipient (n=6)	833.5±70.4	12.0±1.4	195.0±15.2	56.3±8.3

miR-33^−/−^*Apoe*^−/−^ BM recipient (n=8)	984.9±54.7	12.1±1.3	209.9±9.0	43.9±6.6

*P*	NS	NS	NS	NS

Values are mean±SE. BM indicates bone marrow; TC, total cholesterol; TG, triglyceride; LDL-C, low-density lipoprotein cholesterol; HDL-C, high-density lipoprotein cholesterol; NS, not significant.

### MiR-33^+/+^*Apoe*^−/−^ Mice Transplanted With MiR-33^−/−^*Apoe*^−/−^ BM Had Reduced Lipid Accumulation in Atherosclerotic Plaque

After mice were fed a WTD for 12 weeks, atherosclerotic lesions in the proximal aortas were measured. Atherosclerotic plaque formation in mice transplanted with miR-33^−/−^*Apoe*^−/−^ BM tended to be reduced compared with that in mice transplanted with miR-33^+/+^*Apoe*^−/−^ BM, but this difference was not statistically significant (*P*=0.12, 0.53±0.028 versus 0.46±0.033 mm^2^; Figure 10B and 10C). On the other hand, the quantification of lipid accumulation by oil red O staining showed a significant decrease in mice transplanted with miR-33^−/−^*Apoe*^−/−^ BM compared with mice with miR-33^+/+^*Apoe*^−/−^ BM (*P*=0.020, 3.0±0.4% versus 1.9±0.2%; Figure 10D and 10E). The CD68-positive area is shown in Figure 10F and 10G (*P*=0.09, 19.5±2.6% versus 26.6±2.6%). These results showed that loss of miR-33 in blood cells reduced the lipid content of atherosclerotic plaque.

### Free Cholesterol–Induced Apoptosis Was Reduced in PEMs From MiR-33^−/−^*Apoe*^−/−^ Mice Compared With MiR-33^+/+^*Apoe*^−/−^ Mice

**Figure 11.**
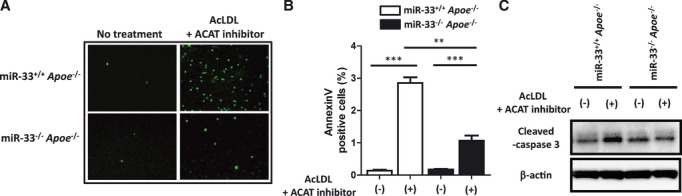
miR-33 deficiency ameliorated free-cholesterol loading-induced macrophage apoptosis. A, Representative microscopic images of Alexa Fluor 448–conjugated annexin V staining. Thioglycollate-elicited peritoneal macrophages from miR-33^+/+^*Apoe*^−/−^ and miR-33^−/−^*Apoe*^−/−^ mice were cultured in the presence or absence of acLDL plus ACAT inhibitor for 24 hours. B, Quantification of annexin V–positive macrophages in the presence or absence of acLDL plus ACAT inhibitor for 24 hours. Values are mean±SE; **P<0.01, ***P<0.001. C, Western blotting analysis of cleaved caspase-3 in macrophages in the presence or absence of acLDL plus ACAT inhibitor for 48 hours. β-actin was used as a loading control.

Because mice transplanted with miR-33^−/−^*Apoe*^−/−^ BM showed reduced lipid accumulation in atherosclerotic plaque compared with mice transplanted with miR-33^+/+^*Apoe*^−/−^ BM, we analyzed free cholesterol (FC)–induced apoptosis in PEMs by treating macrophages with acLDL and acyl-CoA:cholesterol acyl-transferase (ACAT) inhibitor. Figure 11A shows the results of annexin V staining of PEMs with and without acLDL plus ACAT inhibitor treatment. Annexin V–positive cells were significantly increased in PEMs from miR-33^+/+^*Apoe*^−/−^ mice compared with those from miR-33^−/−^*Apoe*^−/−^ mice after FC loading (Figure 11B). Cleaved caspase-3 was also increased in PEMs from miR-33^+/+^*Apoe*^−/−^ mice compared with those from miR-33^−/−^*Apoe*^−/−^ mice after FC loading (Figure 11C).

### MiR-33^−/−^*Apoe*^−/−^ Mice Transplanted With BM of MiR-33^+/+^*Apoe*^−/−^ or MiR-33^−/−^*Apoe*^−/−^ Showed Only a Slight Increase in HDL-C Levels Compared With MiR-33^+/+^*Apoe*^−/−^ Mice Transplanted With BM of MiR-33^+/+^*Apoe*^−/−^ or MiR-33^−/−^*Apoe*^−/−^

**Figure 12.**
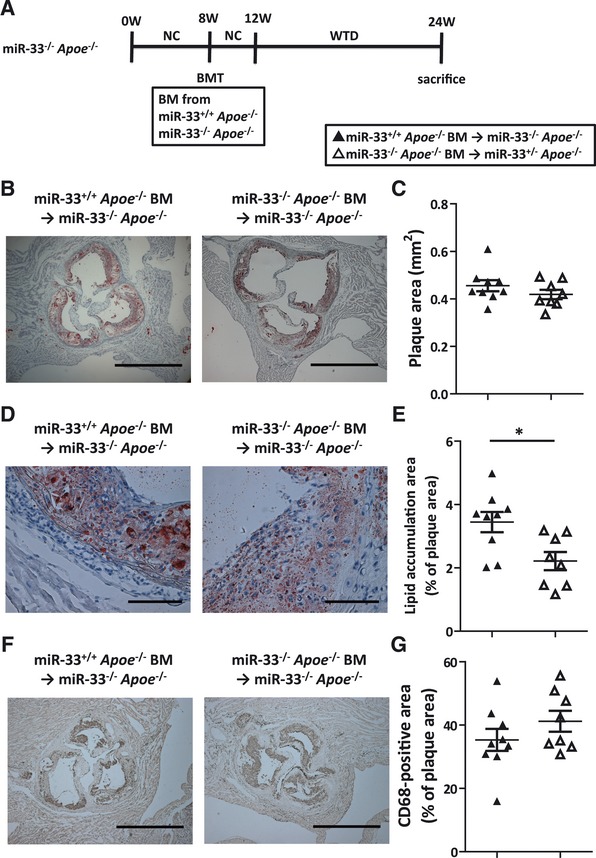
Lipid accumulation area in atherosclerotic lesions was reduced in miR-33^−/−^*Apoe*^−/−^ mice transplanted with miR-33^−/−^*Apoe*^−/−^ BM. A, Experimental protocol for bone marrow transplantation from miR-33^+/+^*Apoe*^−/−^ and miR-33^−/−^*Apoe*^−/−^ mice to miR-33^−/−^*Apoe*^−/−^ mice. B, Representative microscopic images of cross-sections of proximal aorta in mice transplanted with miR-33^+/+^*Apoe*^−/−^ and miR-33^−/−^*Apoe*^−/−^ BM. Scale bar: 1 mm. C, Quantification of the atherosclerotic lesion area in cross-sections of proximal aorta. Values are mean±SE (n=8 to 9 each). D, Representative microscopic images of the lipid accumulation area in atherosclerotic lesions in mice transplanted with miR-33^+/+^*Apoe*^−/−^ and miR-33^−/−^*Apoe*^−/−^ BM. Scale bar: 100 μm. E, Quantification of the lipid accumulation area in cross-sections of proximal aorta. Values are mean±SE (n=8 to 9 each); *P<0.05. F, Representative microscopic images of immunohistochemical staining for the macrophage marker CD68 in mice transplanted with miR-33^+/+^*Apoe*^−/−^ and miR-33^−/−^*Apoe*^−/−^ BM. Scale bar: 1 mm. G, Quantification of the CD68-positive area in cross-sections of proximal aorta. Values are mean±SE (n=8 to 9 each). BM indicates bone marrow.

Previous experiments indicated that loss of miR-33 in blood cells reduced lipid accumulation in atherosclerotic plaque. Next, to determine the contribution of miR-33 deficiency to atherosclerosis in BM recipients, we transferred BM of miR-33^+/+^*Apoe*^−/−^ or miR-33^−/−^*Apoe*^−/−^ mice to miR-33^−/−^*Apoe*^−/−^ mice in the same way as in the previous BMT experiments (Figure 12A). However, the HDL-C levels in miR-33^−/−^*Apoe*^−/−^ mice transplanted with BM of miR-33^+/+^*Apoe*^−/−^ or miR-33^−/−^*Apoe*^−/−^ (Table 3) showed only a slight increase compared with miR-33^+/+^*Apoe*^−/−^ mice transplanted with BM of miR-33^+/+^*Apoe*^−/−^ or miR-33^−/−^*Apoe*^−/−^ (Table 2). Therefore, it was impossible to observe an effect of HDL-C elevation caused by the loss of miR-33 on atherosclerosis in recipients that had the same type of blood cells (Figures 10 and 12).

**Table 3.** Serum Lipid Profiling of MiR-33^−/−^*Apoe*^−/−^ mice transplanted With MiR-33^+/+^*Apoe*^−/−^ and MiR-33^−/−^*Apoe*^−/−^ BM by Standard Method

	TC (mg/dL)	HDL-C (mg/dL)	LDL-C (mg/dL)	TG (mg/dL)
miR-33^+/+^*Apoe*^−/−^ BM recipient (n=9)	875.0±56.0	12.6±1.0	174.6±8.8	108.2±49.0

miR-33^−/−^*Apoe*^−/−^ BM recipient (n=5)	922.8±70.0	13.4±2.1	179.4±12.7	47.2±6.9

*P*	NS	NS	NS	NS

Values are mean±SE. BM indicates bone marrow; TC, total cholesterol; TG, triglyceride; LDL-C, low-density lipoprotein cholesterol; HDL-C, high-density lipoprotein cholesterol; NS, not significant.

### MiR-33^−/−^*Apoe*^−/−^ Mice Transplanted With MiR-33^−/−^*Apoe*^−/−^ BM Had Reduced Lipid Accumulation in Atherosclerotic Plaque

We also observed the effect of loss of miR-33 in BMT experiments in miR-33^−/−^*Apoe*^−/−^ recipients. Atherosclerotic plaque formation in miR-33^−/−^ *Apoe*^−/−^mice transplanted with miR-33^−/−^*Apoe*^−/−^ BM was comparable with that in miR-33^−/−^ *Apoe*^−/−^mice transplanted with miR-33^+/+^ *Apoe*^−/−^ BM (0.46±0.023 versus 0.42±0.020 mm^2^; Figure 12B and 12C). Although the area of lipid accumulation in miR-33^−/−^*Apoe*^−/−^mice transplanted with miR-33^−/−^*Apoe*^−/−^ BM was significantly reduced compared with that in miR-33^−/−^*Apoe*^−/−^mice transplanted with miR-33^+/+^*Apoe*^−/−^ BM (*P*=0.027, 3.4±0.3% versus 2.2±0.3%; Figure 12D and 12E), there was no difference between these mice in the CD68-positive area (*P*=0.37, 35.3±3.5% versus 41.2±3.3%; Figure 12F and 12G).

## Discussion

In the current study, miR-33^−/−^*Apoe*^−/−^ mice showed an increase in HDL-C in vivo and a decrease in atherosclerotic plaque size and lipid content compared with miR-33^+/+^*Apoe*^−/−^ mice. Although a previous study demonstrated that the short-term administration of anti-miR-33 oligonucleotides raised HDL-C levels and promoted the regression of atherosclerosis, this current study indicates that miR-33 deficiency contributes to the reduction of plaque size in the progression of advanced atherosclerosis. Moreover, we assessed the in vivo function of miR-33 deficiency in leukocytes by BMT from miR-33^+/+^*Apoe*^−/−^ or miR-33^−/−^*Apoe*^−/−^ mice into miR-33^+/+^*Apoe*^−/−^ or miR-33^−/−^*Apoe*^−/−^ mice. miR-33^−/−^*Apoe*^−/−^ macrophages promoted the removal of intracellular lipid content compared with miR-33^+/+^*Apoe*^−/−^ macrophages. Together, these data demonstrate that miR-33 deficiency serves to raise HDL-C, improve cholesterol efflux in macrophages, and prevent the progression of atherosclerosis and suggest that miR-33 should be considered as a potential target to prevent the progression of atherosclerosis.

We reported previously that miR-33^−/−^ in C57/BL6 mice increases HDL-C by up to 40%. MiR-33^−/−^*Apoe*^−/−^ mice also had a higher amount of HDL-C compared with controls. However, the static measurement of HDL-C cholesterol level has inherent limitations as a metric of the functional effects of HDL-C in vivo. Moreover, HDL-C function in *Apoe*^−/−^ mice may be altered because these mice had no apoE to activate lecithin:cholesterol acyltransferase, which converts free cholesterol to cholesterol ester, thereby creating a gradient for free cholesterol efflux from cells to HDL-C. Thus, we tried to measure the function of HDL-C from miR-33^−/−^*Apoe*^−/−^ mice. Recently, the functionality of HDL-C was analyzed by the quantification of efflux capacity from blood samples of humans.^25^ Therefore, we measured cholesterol efflux capacity in the serum from miR-33^+/+^*Apoe*^−/−^ and miR-33^−/−^*Apoe*^−/−^ mice. We found that the loss of miR-33 significantly increased the capacity to promote cholesterol efflux, and this may have contributed to the reduction in atherosclerotic plaque volume.

The results of the present BMT experiment revealed that deletion of macrophage miR-33 significantly reduced the lipid content in atherosclerotic plaque. Macrophages are known to ingest apoB-containing lipoproteins and transport ingested lipoprotein-cholesterol from late endosomes to the endoplasmic reticulum (ER) under normal conditions.^41^ In advanced lesional macrophages, the accumulation of massive unesterified, or “free,” cholesterol is observed, which induces ER-stress-mediated macrophage apoptosis.^42,43^ Therefore, we compared atherogenic lipid-induced apoptosis in miR-33^+/+^*Apoe*^−/−^ and miR-33^−/−^*Apoe*^−/−^ macrophages. Treatment of PEMs in culture with acLDL plus ACAT inhibitor (free cholesterol loading) demonstrated that PEMs from miR-33^−/−^*Apoe*^−/−^ mice were significantly resistant to apoptosis compared with those from miR-33^+/+^*Apoe*^−/−^ mice. In accordance with this in vitro experiment, apoptotic cells in the lesion area were also reduced in miR-33^−/−^*Apoe*^−/−^ mice. It is generally considered that apoptosis of macrophages in advanced plaques may lead to plaque rupture. Thus, the suppression of miR-33 in macrophages may also be beneficial for the prevention of plaque rupture.

To further elucidate the effect of miR-33 deletion on the monocyte/macrophage phenotype, we performed a flow-cytometric analysis of circulating monocytes and a quantitative PCR analysis of RNA from PEMs of miR-33^+/+^*Apoe*^−/−^ and miR-33^−/−^*Apoe*^−/−^ mice. We first determined that the total leukocyte count in miR-33^−/−^*Apoe*^−/−^ mice was less than that in miR-33^+/+^*Apoe*^−/−^mice. This was consistent with a previous report that ABCA1, ABCG1, and HDL inhibit the proliferation of hematopoietic stem cells.^44^ Because leukocytosis enhances the progression of atherosclerosis, the reduction in leukocytes observed in miR-33^−/−^*Apoe*^−/−^ mice may have had a beneficial effects on atherosclerosis.^45^ However, we also detected a higher frequency of Ly6C^high^ monocytes in miR-33^−/−^*Apoe*^−/−^ mice than in miR-33^+/+^*Apoe*^−/−^ mice, and this could enhance inflammation in atherosclerotic plaque. Tissue macrophages are phenotypically heterogeneous and are broadly characterized according to their activation (polarization) state by the M1/M2 classification system.^46^ Some M1 and M2 markers were significantly elevated in miR-33^−/−^*Apoe*^−/−^ PEMs compared with miR-33^+/+^*Apoe*^−/−^ PEMs. RIP140 has been reported to promote the activity of NF-κB and to upregulate the expression of genes implicated in inflammation such as TNFα and IL-6 in macrophages.^38^ RIP140 has been shown to be one of the targets of miR-33.^37^ Therefore, the enhanced expression of RIP140 under miR-33 deficiency may have affected the expression of IL-6. It is also possible that elevation of M2 markers may indicate the healing process of atherosclerosis in miR-33^−/−^*Apoe*^−/−^ mice and that the phenotypic changes in macrophages may involve feedback mechanisms. In any case, the effect of miR-33 deletion in macrophages is not as simple as a shift from the M1 to the M2 phenotype, as described in a previous report.^19^ miR-33 deficiency also reduced the expression of VCAM-1, which may have influenced atherosclerotic plaque formation.

We also tried to determine the contribution of the loss of miR-33 in recipient mice to atherosclerosis. However, we did not observe a significant elevation of HDL-C level in miR-33^−/−^*Apoe*^−/−^ mice transplanted with BM of miR-33^+/+^*Apoe*^−/−^ or miR-33^−/−^*Apoe*^−/−^ compared with miR-33^+/+^*Apoe*^−/−^ mice transplanted with BM of miR-33^+/+^*Apoe*^−/−^ or miR-33^−/−^*Apoe*^−/−^. Currently, we do not know why HDL-C levels were almost the same in these mice. Experimental conditions such as radiation to the liver and intestine may have reduced the effect of miR-33 deficiency on the increase in HDL-C levels in recipient mice after BMT. Atherosclerotic plaque size was not significantly reduced in miR-33^−/−^*Apoe*^−/−^ recipients compared with the miR-33^+/+^*Apoe*^−/−^ recipients transferred with the same type of BM; this may simply indicate that the rise in HDL-C levels is important in the prevention of atherosclerosis. Our results showed that loss of miR-33 in blood cells reduced the lipid content of atherosclerotic plaque, which may be because of improved cholesterol efflux from macrophages.

A recent report indicated that the inhibition of miR-33a/b in nonhuman primates raised plasma HDL-C and lowered VLDL triglyceride levels.^47^ This result was obtained from the administration of antisense miR-33 for a certain period. These data suggest that it may be possible to inhibit miR-33a and miR-33b pharmacologically to raise HDL-C for the treatment of dyslipidemia and atherosclerosis. However, to establish the safety of this therapeutic strategy for the treatment of humans, the complete inhibition of target miRNA and longer-term assessment in animal disease models are required to avoid unexpected side effects.

Moreover, the real targets of miR-33 in vivo can only be clarified by the genetic deletion of miR-33, and the results obtained by antisense oligonucleotide-based medicine may be different from those obtained in miR-33-deficient mice. For example, the administration of miR-21 antagomir prevented pressure-overload-induced cardiac hypertrophy and fibrosis in mice^48^; however, miR-21-deficient mice did not show any cardiac difference with wild-type mice under pressure overload.^49^ There also seems to be a substantial difference in the effect of antisense oligonucleotide against miRNA depending on the modification such as LNA based or cholesterol modified, and many additional miRNAs that share an identical or similar sequence may be inactivated. Therefore, caution is needed when interpreting studies that use antagomir approaches to elucidate the function of individual miRNAs in vivo. Previously, we and others showed that miR-33 targets ABCA1 in macrophages and the liver. ABCG1 is another target in macrophages. In this study, we showed that ABCA1 and CROT in the livers of miR-33^−/−^*Apoe*^−/−^ mice were upregulated compared with those in miR-33^+/+^*Apoe*^−/−^ mice, whereas CPT1a and AMPKα were not. ABCA1, ABCG1, and RIP140 were also upregulated in miR-33^−/−^*Apoe*^−/−^ macrophages. Thus, although many targets have been estimated by many computer algorithms and in vitro experiments such as luciferase-based 3′ UTR analysis and Western blotting, it seems that only some of these actually have any effect in vivo through the chronic complete inhibition of miRNA.

Metabolic syndrome and type 2 diabetes are growing public health concerns worldwide that are associated with complex risk factors for cardiovascular disease (CVD). A previous meta-analysis indicated that a reduction of 2 to 3 mmol/L LDL-C by statins could reduce the risk of CVD by 40% to 50% because there was no evidence of any threshold within the cholesterol range studied.^3^ However, this simply means that there is still a substantial risk of CVD despite statins being widely used to lower levels of LDL-C and apolipoprotein B–containing lipoproteins. Major goals in the pursuit of novel therapeutic strategies to target this residual risk have focused on raising the amount and quality of HDL-C to prevent atherosclerosis. Further detailed experiments will be needed to determine whether targeting miR-33 could be a suitable approach for such treatment.
